# Anti‐inflammatory effects of infliximab in mice are independent of tumour necrosis factor α neutralization

**DOI:** 10.1111/cei.12872

**Published:** 2016-11-23

**Authors:** B. M. Assas, S. E. Levison, M. Little, H. England, L. Battrick, J. Bagnall, J. T. McLaughlin, P. Paszek, K. J. Else, J. L. Pennock

**Affiliations:** ^1^ Faculty of Applied Medical SciencesKing AbdulAziz UniversityJeddahSaudi Arabia; ^2^ Faculty of Biology Medicine and HealthUniversity of ManchesterManchester; ^3^ Manchester Royal InfirmaryManchesterUK; ^4^ School of Biological Sciences, Faculty of Medicine Biology and HealthUniversity of ManchesterManchesterUK

**Keywords:** antibody, inflammation, macrophage, mucosa

## Abstract

Infliximab (IFX) has been used repeatedly in mouse preclinical models with associated claims that anti‐inflammatory effects are due to inhibition of mouse tumour necrosis factor (TNF)‐α. However, the mechanism of action in mice remains unclear. In this study, the binding specificity of IFX for mouse TNF‐α was investigated *ex vivo* using enzyme‐linked immunosorbent assay (ELISA), flow cytometry and Western blot. Infliximab (IFX) did not bind directly to soluble or membrane‐bound mouse TNF‐α nor did it have any effect on TNF‐α‐induced nuclear factor kappa B (NF‐κB) stimulation in mouse fibroblasts. The efficacy of IFX treatment was then investigated *in vivo* using a TNF‐α‐independent *Trichuris muris‐*induced infection model of chronic colitis. Infection provoked severe transmural colonic inflammation by day 35 post‐infection. Colonic pathology, macrophage phenotype and cell death were determined. As predicted from the *in‐vitro* data, *in‐vivo* treatment of *T. muris‐*infected mice with IFX had no effect on clinical outcome, nor did it affect macrophage cell phenotype or number. IFX enhanced apoptosis of colonic immune cells significantly, likely to be driven by a direct effect of the humanized antibody itself. We have demonstrated that although IFX does not bind directly to TNF‐α, observed anti‐inflammatory effects in other mouse models may be through host cell apoptosis. We suggest that more careful consideration of xenogeneic responses should be made when utilizing IFX in preclinical models.

## Introduction

Infliximab (IFX) is a neutralizing monoclonal chimeric antibody first developed against human tumour necrosis factor (TNF)‐α in BALB/c mice 1 and is used to treat a variety of chronic inflammatory disorders, including Crohn's disease. To reduce immunogenicity the light and heavy chains from the variable region of the original hybridoma antibody were joined to human kappa and immunoglobulin (Ig)G1 constant regions. Several studies have suggested that the efficacy of IFX in humans cannot be accounted for by TNF‐α neutralization alone 2, 3, and various mechanisms have been suggested.

Preclinical models add value to understanding the complex mechanisms of cytokine neutralization, and since 2012 more than 25 studies have demonstrated an anti‐inflammatory effect of IFX treatment in mice, with an inferred mechanism of TNF‐α neutralization. Several studies have shown an overall reduction in TNF‐α after treatment 4, 5, 6. However, it has never been shown convincingly that IFX binds mouse TNF‐α or whether xenogeneic responses play a role, despite initial claims that IFX has no cross‐reactivity with mouse or rat 1.

We were interested to examine the specificity of IFX for mouse TNF‐α in more detail to understand the value of IFX in preclinical mouse models. Here we used both *in‐vitro* and *in‐vivo* approaches to study the specific effects of IFX in mice. We used a TNF‐α‐independent infection‐driven mouse model of chronic colitis 7, 8. Chronic *Trichuris muris* infection presents with transmural colonic inflammation, a dominant T helper type 1 (Th1) response and inflammatory cell influx. However, it has been shown that the lack of receptors for both TNF‐α 9 and TNF‐α neutralization 8 does not change the clinical outcome of chronic infection. The inflammatory colitic phenotype driven by TNF‐α‐independent mechanisms made this model ideal to study IFX treatment in mice, as any observed phenotype post‐IFX treatment must have a basis in a non‐TNF‐α neutralization mechanism.

## Methods

### Animals

Six‐week‐old male AKR mice (Harlan Olac Ltd, Bicester, UK) were housed under specific pathogen‐free conditions with free access to food and water. TNF‐α‐deficient mice (Charles River, Oxford, UK) were maintained and bred on a C57BL/6 background at the University of Manchester. All experiments were conducted under the UK Home Office Animals Scientific Procedures Act 1986 (revised January 2013) at the University of Manchester.

### Western blot

Binding of IFX to recombinant (r)TNF‐α was studied using standard denaturing Western blot techniques. Briefly, 2‐mercaptoethanol and heat‐treated rTNF‐α was loaded onto a preprepared iBlot sodium dodecyl sulphate (SDS) denaturing gel (Novex; Life Technologies, Paisley, UK) in denaturing SDS running buffer (NuPAGE Tris‐Acetate SDS; Invitrogen Life Technologies). After transferring to a nitrocellulose membrane, the gel was blocked (5% fish gelatin for 1 h, shaking), washed [1% phosphate‐buffered saline (PBS)‐Tween20 ×4] and primary antibodies added [infliximab, 2 μg/ml; polyclonal rabbit anti‐mouse TNF‐α (Abcam ab9739; Abcam, Cambridge, UK), 10 μg/ml]. Membranes were incubated for 16 h at 4°C, washed and secondary antibodies [rabbit anti‐human IgG, 1 μg/ml (Abcam); goat anti‐rabbit, 2 μg/ml (Abcam)] were added (1 h with shaking). Membranes were then washed and treated with ECL (1 min; Promega, Southampton, UK) before imaging using Bio‐Rad Chemidoc (Bio‐rad, Hertfordshire, UK). Data were processed using Image Lab Software (Bio‐rad).

### Spleen cell assays

Spleen cells were isolated using standard techniques. After red blood cell lysis cells were washed and plated in RPMI‐1640 complete media [10% fetal calf serum (FCS), 2 mM L glutamine, 100 U/ml penicillin and 100 μg/ml streptomycin] at 5 × 10^5^ cells/well. Cells were stimulated with lipopolysaccharide (LPS) (50 μg/ml) in triplicate, with or without IFX (20 μg/ml). Cells were incubated at 37°C/5% CO_2_ for 24 h. Transmembrane tmTNF‐α was detected by flow cytometry (BD Pharmingen clone MP6‐XT22, 1 μg/ml; BD Pharmingen, Oxford, UK). Recommended isotype control and TNF‐α‐deficient mice were used as a negative control. Cells were stained separately for cell death markers propidium iodide [PI‐phycoerythrin (PE)] and annexin V [AV‐fluorescein isothiocyanate (FITC)], according to the manufacturer's instructions (eBioscience, Altrincham, UK).

### Luminometry

Mouse embryonic fibroblasts stably expressing a luciferase‐labelled consensus nuclear factor kappa B (NF‐κB) promoter (generated by lentiviral transduction) was constructed. Lentiviral luciferase reporter for NF‐κB transcriptional activity was made by insertion of a multiple cloning site into pRE1x plasmid (a kind gift from U. Shibler) 31 using the ecoRV and age1 restriction enzymes. Subsequently, a 5× repeat κB consensus and minimal promoter element was cloned using the restriction enzymes *pac1* and *nhe1*. Lentivirus production and transduction was carried out as described previously 32. Transduced cells were plated in medium containing luciferin (0·5 mM; Biosynth AG, Staad, Switzerland) and mouse rTNF‐α (50 μg/ml), IFX, anti‐mouse TNF‐α, hu or rat IgG (100 μg/ml, 10 μg/ml or 1 μg/ml) was added to the media. Live cell measurements were made using the FLUOstar Omega plate reader (BMG Labtech, Aylesbury, UK) with incubation at 37°C and 5% CO_2_. Wells were measured every 10 min during a 12‐h period. Data were normalized to an untreated control.

### Induction of experimental colitis and treatment

*Trichuris muris* parasites were harvested and maintained as described previously 7. Infected AKR mice received 300 embryonated *T. muris* eggs in distilled water by oral gavage. A single injection of 5 mg/kg (paediatric dose) of infliximab (Remicade, MSD, Hoddesdon, UK), was given intraperitoneally (carrier 0·9% sodium chloride) 35 days post‐infection. Control antibodies were given at the same concentration; anti‐mouse TNF‐α (XT22, rat anti‐mouse IgG1; eBioscience), rat IgG1 or human IgG (Sigma Aldrich, Poole, UK).

### Phenotyping

Serum samples, mesenteric lymph node (MLN) and intestines were taken at autopsy. The colon was measured from ileo‐caecal valve to rectal margin; 0·5 cm of whole colonic tissue immediately distal to the ileo‐caecal valve was isolated into 1·0 ml AllProtect^®^ (Qiagen, Manchester, UK) for 24 h (4°C) before RNA extraction. For histology, 0·5 cm of whole colonic segments from the proximal ascending colon were taken and fixed in neutral buffered formalin. The remaining large bowel was assessed for worm burden.

### Histological evaluation

5 μm paraffin‐embedded sections were stained with Harris haematoxylin and eosin using standard protocols. Slides were randomized and blinded. Colonic crypt length and muscle thickness (μm) were measured using Image J software (http://rsbweb.nih.gov/ij) following image capture using SPOT™ Imaging Solutions camera and Advanced SPOT software (Diagnostic Instruments Inc., Sterling Heights, MI, USA). Terminal deoxynucleotidyl transferase dUTP nick end labelling (TUNEL) staining was performed on 5 μm paraffin sections, according to the manufacturer's instructions (*in situ*‐cell death detection kit; (Roche) Sigma Aldrich).

### Statistical analysis

Statistical testing was performed using GraphPad Prism^®^ version 5.00 (GraphPad Software, Inc., San Diego, CA, USA), with one‐way analysis of variance (anova) and unpaired Student's *t*‐test *post‐hoc* analysis. Data are expressed as mean ± standard error of the mean (s.e.m.).

## Results

### Infliximab does not bind mouse TNF‐α

**Figure 1:**
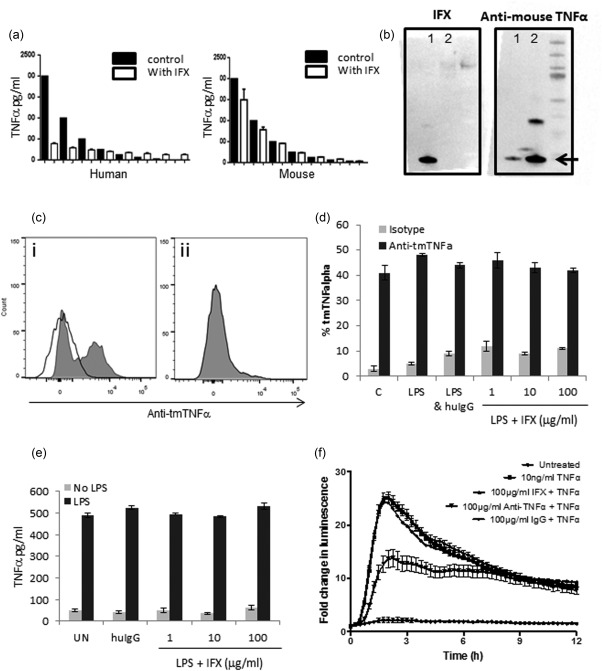
Infliximab (IFX) does not bind to soluble or membrane‐bound mouse tumour necrosis factor (TNF)‐α. **(**a) Competitive binding assay. IFX (20 μg/ml) blocked human recombinant (r)TNF‐α binding in an enzyme‐linked immunosorbent assay (ELISA) assay, but did not block mouse rTNF‐α binding in the equivalent mouse ELISA. (b) Detection of human (lane 1) or mouse (lane 2) rTNF‐α by Western blot. First gel: infliximab was able to detect human (lane 1) but not mouse TNF‐α (lane 2) when used as a primary detection antibody. Second gel: rat anti‐mouse TNF‐α detected mouse rTNF‐α and showed some cross‐reactivity with human rTNF‐α (lane 1). (c) Positive staining of C57BL/6 splenocytes (i) with anti‐TNF‐α antibody (solid grey peak) by flow cytometry, compared to isotype control (solid line). TNF‐α‐deficient mice (ii) are shown as controls. (d) Quantification of tmTNF‐α detection by flow cytometry after treatment with lipopolysaccharide (LPS), LPS and IFX or human innunoglobulin (Ig)G control. (e) IFX does not block sTNF‐α production from RAW293 macrophages stimulated with LPS (50 μg/ml) *in vitro*. TNF‐α detected by ELISA. (f) Luminometry trace showing no effect of IFX on nuclear factor kappa B (NF‐κB) signalling in response to TNF‐α in transfected mouse embryonic fibroblast (MEF) cells. Peak represents translocation of NF‐κB to the nucleus. (a–e) Data representative of three independent experiments (*n* = 3–5). (f) Data representative of one experiment run in triplicate. Data shown as mean +/–standard error of the mean.

We set out initially to determine the binding capacity of IFX to both soluble (sTNF‐α) and membrane‐bound (tmTNF‐α) mouse TNF‐α. Although IFX was able to block binding of anti‐human TNF‐α antibody to recombinant human TNF‐α by ELISA, it had no equivalent effect in a mouse TNF‐α ELISA (Fig. 1a). This was confirmed by Western blot (Fig. 1b), revealing an inability of IFX to recognize mouse rTNF‐α. It has been suggested that IFX binds the human membrane‐bound trimer of tmTNF‐α with high affinity 10, so we sought to determine whether IFX could block the detection of murine tmTNF‐α by flow cytometry. Figure 1c shows binding of anti‐mouse TNF‐α antibody by flow cytometry in C57BL/6 (wild‐type, Fig. 1ci) and TNF‐α‐deficient mice as controls (Fig. 1cii). IFX did not block detection of tmTNF‐α by flow cytometry at either 1, 10 or 100 μg/ml (Fig. 1d) after treatment of whole spleen cells with LPS. Furthermore, treatment with IFX during LPS stimulation had no effect on production or detection of sTNF‐α in the supernatant by ELISA (Fig. 1e). Finally, to confirm the lack of mouse TNF‐α epitope blocking, mouse embryonic fibroblasts loaded with luciferase‐labelled NF‐κB were incubated with sTNF‐α with or without IFX, control human IgG or anti‐TNF‐α antibody. Luminescence was measured over 12 h (Fig. 1f). Luminometry demonstrated very clearly that unlike anti‐mouse TNF‐α antibody, neither IFX nor human IgG inhibited the activation of NF‐κB in mouse fibroblasts in response to TNF‐α. IFX was titrated from 1 to 100 μg/ml; no effect on NF‐κB expression was observed at any concentration (data not shown).

### Infliximab treatment did not affect the clinical outcome of *T. muris‐*induced chronic colitis

**Figure 2:**
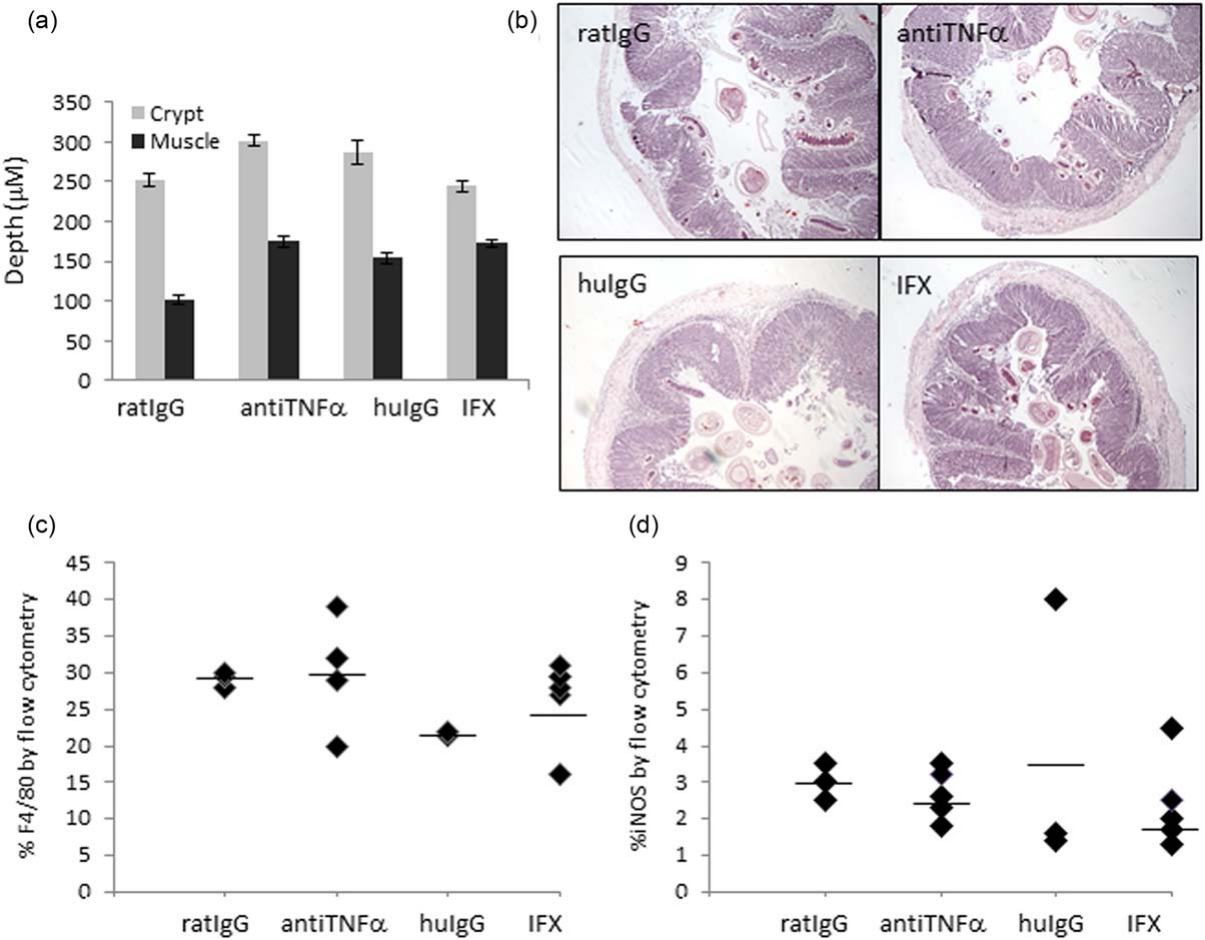
Infliximab (IFX) treatment has no effect on clinical outcome of chronic *Trichuris muris* infection in AKR mice. **(**a) Infection‐induced crypt hyperplasia was not affected by control or antibody [IFX/anti‐tumour necrosis factor (TNF)‐α] treatments. (b) Representative haematoxylin and eosin‐stained colonic sections of experimental groups showing no significant difference in macroscopic pathology after treatment. (c,d) Flow cytometry of isolated colonic cell suspensions stained with F4/80 (c) and inducible nitric oxide synthase (iNOS) (d) to define macrophage populations. No significant effect of treatment on macrophage number was seen. Data represented as mean +/–standard error of the mean; *n* = 3–5 per group. Data representative of two independent experiments. [Colour figure can be viewed at wileyonlinelibrary.com]

As IFX has been shown to induce anti‐inflammatory effects in mice, we chose to measure efficacy in a TNF‐α‐independent model of colitis, induced by a natural pathogen of mice *T. muris* 7, 11. Mice were studied alongside a neutralizing anti‐mouse TNF‐α antibody or human/mouse IgG treatments as controls. Treatments were administered day 35 post‐infection when chronic inflammation was established. No significant differences were seen between infected groups in either worm burden (193 ± s.e.m.) or weight loss (data not shown). Altered stool pellets, gross inflammatory changes (oedema, colonic tissue texture) and colonic shortening were seen in all infected mice. Histologically, treatments had no significant effect on infection‐induced crypt hyperplasia (Fig. 2a). Mild‐to‐moderate inflammatory changes were observed in all infected groups, including transmural tissue oedema, leucocytic infiltration (lymphocytes, macrophages, neutrophils), prominent mucosal and submucosal reactive lymphoid aggregates (Fig. 2b). Neither anti‐TNF‐α treatment or IFX exerted any effect on naive mice (data not shown).

### Infliximab did not reduce colonic macrophage influx in *T. muris‐*infected colitic mice

As both soluble and tmTNF‐α plays a key role in macrophage recruitment and function during inflammation, we isolated lamina propria cells from all groups to analyse macrophage phenotype and number by flow cytometry. There was no significant difference in F480^+^ or inducible nitric oxide synthase (iNOS)^+^ cells after treatment, suggesting that IFX did not affect macrophage survival or influx in the short 10‐day window post‐treatment (Fig. 2c,d). Similarly, parallel treatment of *T. muris‐*infected mice with anti‐mouse TNF‐α monoclonal antibody or associated control antibody demonstrated that in the *T. muris* infected mouse, macrophage number is unaffected by either human antibody, TNF‐α neutralization or IFX treatment (Fig. 2c,d).

### Infliximab induced significant cell death in mice both *in vitro* and *in vivo*

**Figure 3:**
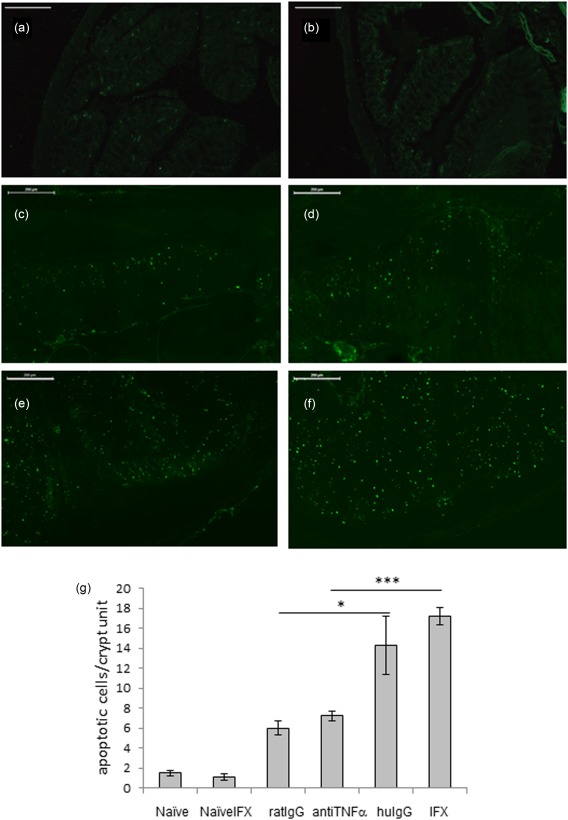
Infliximab and human immunoglobulin (Ig)G cause significant apoptosis in *Trichuris muris‐*infected mice. Colitic mice were treated with infliximab (IFX), and assessed for apoptotic cells [terminal deoxynucleotidyl transferase dUTP nick end labelling‐fluorescein isothiocyanate (TUNEL‐FITC)] in proximal colon 10 days after single treatment day 35 post‐infection. (a) Uninfected untreated; (b) uninfected IFX‐treated; (c) infected rat IgG‐treated; (d) infected anti‐mouse tumour necrosis factor (TNF)‐α; (e) infected human IgG‐treated; (f) infected IFX‐treated. Micrographs representative of two independent experiments; *n* = 5 in each group. (a,b) Scale bar 500 μm; (c–f) scale bar 200 μm. [Colour figure can be viewed at wileyonlinelibrary.com]

*In vivo*, both IFX and human IgG treatment induced significant cell cytotoxicity (by TUNEL staining) in the proximal colon at the site of infection, primarily at mucosal and submucosal sites, clearly indicating a systemic effect of the human antibody administration (Fig. 3). A highly significant increase in apoptosis was observed in infected IFX‐treated mice compared to the anti‐mouse TNF‐α‐treated group (*P* = 5 × 10^−5^), suggesting that IFX mediated its effect on cell survival independently of TNF‐α, a conclusion reinforced by the similar effect of human IgG. Very little cell apoptosis was observed at the epithelial interface; the most abundant cell death was observed in the base of the crypts for all treatments.

**Figure 4:**
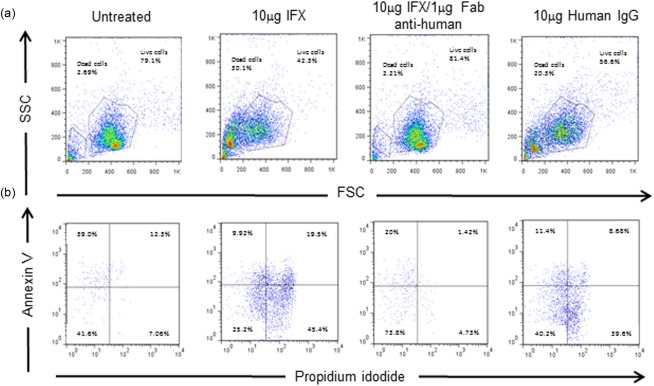
Infliximab and human immunoglobulin (Ig)G induce cell death in mice *in vitro*. (a) Whole splenocytes were cultured with/out infliximab alongside control human IgG and blocking anti‐human Fc antibody (Fab fragment). (b) Cells were stained for propidium iodide [phycoerythrin (PE)] uptake and annexin V expression [fluorescein isothiocyanate (FITC)] and analysed by flow cytometry. Data representative of three independent experiments. [Colour figure can be viewed at wileyonlinelibrary.com]

It has been shown *in vitro* that IFX can inhibit the TNF‐R1 : TNF‐α reverse signalling pathway between T cells and macrophages, affecting cell survival 12. We therefore consolidated the *in‐vivo* observations by culturing whole spleen cells with or without IFX, human IgG1 and/or anti‐human Fc antibody. IFX and human IgG control treatment induced significant cell death in whole spleen cell populations over a 24‐h period; both apoptotic and necrotic cells could be seen (Fig. 4). Blocking of IFX with anti‐human Fc IgG (Fab fragment) blocked the death observed, suggesting a role for the Fc portion of IFX in cytotoxicity.

## Discussion

Since 2012 many peer‐reviewed papers have studied the effect of IFX in mouse models of inflammation, from diabetes 13 and colon cancer 14 to colitis 15, 16, 17. The use of preclinical models to investigate the mechanism of action and efficacy of therapeutic agents is key to drug development. However, despite initial claims that IFX does not cross‐react with mouse, rat, dog or pig TNF‐α^1^, IFX has continued to be used in mouse preclinical models. Contrary to a recent report 18, we have shown using three independent techniques (ELISA, Western blot and flow cytometry) that IFX does not bind mouse TNF‐α. One previous study has shown that IFX does not bind mouse rTNF‐α by ELISA, but no significant conclusions about preclinical models were drawn 19. We show here that, functionally, IFX does not prevent TNF‐α production by LPS‐stimulated spleen cells, nor does it block NF‐κB signalling in mouse fibroblasts induced by soluble TNF‐α. IFX has been shown previously to increase apoptosis in an inflammatory environment in mice 20, and our data support this work. It has been suggested that IFX induces T cell apoptosis specifically in Crohn's patients through binding to tmTNF‐α 3, removing the key reverse signalling survival pathway for both macrophages and T cells inducing significant apoptosis. It has also been shown that IFX can inhibit intestinal epithelial cell apoptosis in both mice 21 and humans 16, protecting enteric barrier integrity. Both these mechanisms rely in part on binding to TNF‐α. Here we have demonstrated that in mice these inferred mechanisms are independent of specific TNF‐α neutralization. We suggest instead that IFX drives anti‐inflammatory effects in mice through an Fc‐mediated mechanism.

The mechanism of action of IFX outside TNF‐α binding has been the subject of many studies over the years, concluding that its efficacy in humans cannot be accounted for by TNF‐α neutralization alone 2. Clearly, our data do not contest these observations, but strongly bring into question investigation of these mechanisms in preclinical mouse models. Similarly, caution must be exercised when concluding that a positive clinical outcome after treatment in mice confers a key role for TNF‐α in the model in question. For example, while IFX has been shown to reduce pathology and disease activity in the dextran sulphate sodium (DSS) colitis model 18, neither transcriptional inhibition of TNF‐α 22 nor genetic deficiency of TNF‐α has been shown to increase susceptibility 23. Even in cases where a model specifically uses mice transgenic for human TNF‐α 24, 25, alternative approaches to TNF‐α neutralization should be considered alongside. For instance, Biton *et al*. compared both IFX and TNF‐α kinoid treatment in arthritic human TNF‐α transgenic mice, showing that both treatments had the same immunological outcome 26. Use of appropriate alternatives and controls is particularly important for models of diseases where anti‐TNF‐α therapies are used clinically, such as rheumatological and inflammatory bowel diseases. There are no publications, for instance, which show convincingly that etanercept can specifically bind mouse TNF receptor, despite an observed effect of treatment on clinical outcome 27, 28.

In summary, it is clear that in many murine models of inflammation a consequence of IFX treatment is reduction in TNF‐α 6, 21, 29. However, we assert that this is not due to direct inhibition of TNF‐α production or signalling, but rather is more likely to be due to cell loss and the natural immunogenicity of IFX in mice. The data presented here do not refute observations with IFX treatment made in mice to date, but rather suggest revisiting each of the inflammation models to determine the true role of TNF‐α in disease pathogenicity. These data also support strongly the use of humanized or human–chimeric mice for future preclinical therapeutic study 30.

## Author contributions

S. E. L., J. T. M., P. P., K. J. E. and J. L. P.: study concept and design. M. B. A., S. E. L., M. L., H. E., L. B. and J. B.: acquisition of data. S. L., M. B. A., M. L., H. E., K. J. E. and J. L. P.: analysis and interpretation of data. M. B. A., K. J. E. and J. L. P. drafting of the manuscript.

## Disclosure

The authors declare no disclosures, financial or otherwise in relation to this work.
